# Leveraging latent assets to strengthen the COVID-19 response and vaccine roll-out in Africa

**DOI:** 10.1136/bmjgh-2021-006289

**Published:** 2021-05-27

**Authors:** Neema Kaseje, Kennedy Oruenjo, Dan Kaseje, Timothy Grant Evans, Marcel Tanner, Andy Haines, James Nyikal

**Affiliations:** 1 Surgical Systems Research Group, Kisumu, Kenya; 2 Siaya Ministry of Health, Siaya, Kenya; 3 Tropical Institute of Community Health and Development, Kisumu, Kenya; 4 McGill University, Montreal, Quebec, Canada; 5 Swiss Tropical and Public Health Institute, Basel, Switzerland; 6 London School of Hygiene and Tropical Medicine, London, UK; 7 Parliament of the Republic of Kenya, Nairobi, Kenya

**Keywords:** COVID-19, vaccines

**Summary box**

- Countries in sub-Saharan Africa have faced challenges in COVID-19 vaccine roll-out, including the lack of infrastructure needed to maintain cold chains, inadequate vaccine supplies, growing vaccine hesitancy and the lack of funding.
- Our experience implementing an integrated intervention leveraging the community health strategy, youth, and digital technology to maximize COVID-19 prevention and optimize COVID-19 case management in communities and health facilities demonstrated key assets that are critical to strengthening the COVID-19 response and vaccine rollout in Africa, namely, (1) community-based healthcare (CBHC) with community health workers taking the lead; (2) youth engagement: in our intervention in Siaya, we included youth to promote digitising household visits for rapid access to household-level data; and (3) social capital: in rural areas of Africa like Siaya, social capital represents a rich resource. As a result of actively engaging with communities, we observed high adherence with non-pharmaceutical interventions.
- As vaccines become more available in Africa through local production and platforms such as COVAX, let us not forget assets present in Africa that can accelerate the COVID-19 vaccine roll-out, enhancing the prevention package: CBHC, youth and social capital.

There has been a recent focus on challenges African countries will encounter during the roll-out of COVID-19 vaccines, including the lack of infrastructure needed to maintain cold chains, inadequate vaccine supplies, growing vaccine hesitancy and the lack of funding.^1^

However, rather than focusing solely on challenges, we argue African countries should recognise and harness assets that could accelerate the vaccine roll-out and enhance the COVID-19 prevention package.^2^ This view is based on our experience in Siaya, where we implemented an integrated intervention leveraging the community health strategy, youth and digital technology to maximise COVID-19 prevention and optimise the case management of patients with COVID-19 in communities and health facilities.^3^

In Siaya (rural Kenya), following a baseline assessment of pandemic preparedness which revealed gaps in COVID-19 training and basic equipment including pulse oximeters, we implemented an integrated intervention that prioritised healthcare worker training in infection prevention and control measures, contact tracing, continued availability and use of essential health services, and case management of patients with COVID-19 in communities and health facilities using pulse oximeters.^3^ Youth were involved in promoting digital household visits and data collection. Following the intervention, we demonstrated a close to eightfold reduction in COVID-19 cases compared with neighbouring counties; furthemore, we observed a reduction in diarrhoeal cases and upper respiratory tract infections not related to COVID-19 as a result of improved hand hygiene.^3^ Unlike other pandemic responses where reductions in the use of essential health services including maternal health services were observed, in Siaya, the use of maternal health services was maintained during the COVID-19 response.^3^

The Siaya project success links to three critical assets in Africa:

1. Community-based healthcare (CBHC) with community health workers (CHWs) taking the lead: CHWs link households to the health system. In our experience during the COVID-19 response, CHWs were critical in sensitising community members about COVID-19 prevention and home- based care. As a result, we observed high household adherence to mask wearing and handwashing and later a high vaccine acceptance rate. During our last survey of 23 452 households visited, 84.1% of households had adequate mask wearing, 86% of households had adequate handwashing facilities and 90.7% of households would accept the COVID-19 vaccine if offered. This vaccine acceptance rate is higher than recent global estimates ranging from 40% to 55%^4^

CBHC has been used as a strategy to deliver essential health services in many parts of Africa.^5^ Africa can leverage this asset to maximise the uptake of COVID-19 vaccines, when available, to enhance and not displace the current prevention package of mask wearing, handwashing and physical distancing. Furthermore, as we observed in Siaya, CBHC can be a mechanism for building and maintaining trust. We need communities on our side as we confront multiple waves of COVID-19 infections and prepare for future pandemics.

1. Youth engagement: In our intervention in Siaya, we included youth to promote digitising household visits for rapid access to household-level data. We demonstrated the feasibility of including youth in health interventions during a pandemic. Africa as the youngest continent can better leverage its youth during health interventions to fully harness the digitisation process that has been accelerated by the COVID-19 pandemic. Furthermore, youth can be a formidable voice to counter misinformation on social media and other platforms with verified health information.
2. Social capital: In rural areas of Africa like Siaya, social capital represents a rich resource. As a result of actively engaging with communities, we observed high adherence with non-pharmaceutical interventions. Moreover, to date, we have not observed community members protesting masking mandates on account of individual rights. With vaccine access still far from being universal and the prospect of multiple waves of infections, the need for sustained adherence to public health measures is indispensable.

African countries can take advantage of the social capital currently in place to maintain the foundation of the prevention package (mask wearing, handwashing and distancing) so that schools can remain open, and a minimum level of economic activity can continue to sustain dignified livelihoods.

CBHC and CHWs have played critical roles in pandemics globally. A recent systematic review summarised evidence from 17 countries in low/middle-income countries (14 from sub-Saharan Africa and 3 from Asia) and 3 from high-income countries.^6^ In addition to documenting the critical roles CHWs play in community awareness, engagement, sensitisation, contact tracing, surveillance and data collection, they highlighted a need for CHW training in pandemic preparedness, more clarity in the definition of essential activities and recognised the need for more research in this area.^6^

In Siaya, we built additional evidence on the role of CHWs in pandemics.^3^ We prioritised healthcare worker training in pandemic preparedness at community and health facility levels.^3^ Essential activities focused on maximising COVID-19 prevention and maintaining access to essential health services.^3^ Furthermore, we demonstrated the feasibility of engaging youth in health interventions during a pandemic.^3^ This is a new approach that can be transformational in countries with young populations. Finally, we recognised the benefits of social capital in promoting collective adherence to mask wearing and handwashing.

As vaccines become more available in Africa through local production and platforms such as COVAX, let us not forget assets present in Africa that can accelerate the COVID-19 vaccine roll-out, enhancing the prevention package: CBHC, youth and social capital.

## Data Availability

All data relevant to the study are included in the article.
